# Knowledge, attitudes and practices of the liberal doctors in relation to the national convention signed in the framework of Mandatory Health Insurance in Morocco: a cross-sectional study

**DOI:** 10.11604/pamj.2018.29.139.14407

**Published:** 2018-03-02

**Authors:** Soufiane Zegraoui, Amine Cheikh, Mustapha Bouatia, Mohamed Rida Ajaja, Saida Naji, Amine El Hassani

**Affiliations:** 1 Mohammed V University, Faculty of Law and Economics, Rabat, Morocco; 2 Abulcasis University, Faculty of Pharmacy, Cheikh Zaid Hospital, Rabat, Morocco; 3 Mohammed V University, Faculty of Medicine and Pharmacy, Pediatrics Hospital, Rabat, Morocco; 4 Abulcasis University, Faculty of Medicine, Cheikh Zaid Hospital, Rabat, Morocco; 5 Mohammed V University, Faculty of Medicine and Pharmacy, Cheikh Zaid Hospital, Rabat, Morocco

**Keywords:** Mandatory Health Insurance, National Convention, liberal doctors

## Abstract

**Introduction:**

under the Mandatory Health Insurance (MHI) scheme, liberal doctors signed their first national convention in the year of 2006. The delay in renewing this agreement could negatively affect the accessibility of the insured persons to medical care. The objective of this study was to explore the knowledge, attitude and practice of the liberal doctors towards their adherence to the national convention signed under MHI scheme and to propose some improvements.

**Methods:**

our study is cross-sectional based on a descriptive survey targeting the population of liberal doctors adhering to the signed convention under the MHI in Morocco. The material used was a questionnaire that was administered to doctors selected. The processing and analyzing of results were performed with SPSS 13.0.

**Results:**

the study, conducted in 2016, examines included 40 liberal doctors. 97.5% of them reported dissatisfaction with the National Reference Pricing. 60% of the them were demotivated to the application of the national convention because of the lack in educational materials. There was no significant difference in the attitudes between general practitioners and specialists, all of whom considered that remuneration was unfair under MHI (p = 0.689), they also considered that working conditions have deteriorated (p = 0.256).

**Conclusion:**

the behavior of liberal doctors towards the national convention signed within the framework of the MHI hides a general dissatisfaction whatever the place of practice. This dissatisfaction was felt by physicians regardless of their seniority or specialty. Several efforts should be made to find a compromise between doctors and the health insurance system to improve patient access to care.

## Introduction

Conventions are defined as a set of coordination rules, respected with a high degree of regularity or are followed because they are simply self-evident [1]. They come to solve small and large problems of coordination of economic and social life [1]. The national convention, linking the MHI management bodies on the one hand and the health professionals on the other hand, is an effective regulatory instrument that can significantly reduce the costs of patient health care in the private sector [2]. The national convention is therefore necessary to control the costs of health services [2]. It allows setting the tariffs of the fees that will serve as the basis of reimbursements for the health insurance funds [2]. According to the Moroccan law of a basic insurance health (Law 65-00), the national convention is a contract between the managing bodies of MHI on the one hand, and providers of medical goods and services on the other hand, under the aegis of the National Agency of Health Insurance (NAHI), which is responsible for conducting the negotiations relating to the establishment of this convention [3]. The purpose of a national convention is thus to determine the rights and obligations of both parties (insurance funds and private doctors) by setting a National Reference Tariff (NRT) for medical services [3]. Since the application of MHI in 2006, the National Convention has provided considerable support for improving public health spending, improving access to medicines and reducing inequities in access to care between the two public and private sectors [4]. However, at a time when the reimbursement rates and the health care basket improved, NRF remained virtually stable [5]. This reality weighs heavily on co-payment borne by MHI policyholders. The strategy of health in Morocco covering the period 2012-2016 aimed to reduce direct house-hold expenditure from 53% to 25% [6]. At the end of 2016, these expenses remained at around 50%, thus exposing patients to a risk of impoverishment in the event of illness [5]. The cost of health care is growing more rapidly in the private sector than in the public sector [5]. With the stability of the NRT, this evolution could lead to the violation of the terms of the national convention, in particular by the liberal doctors. A situation which deserves much attention from the Ministry of Health and especially with the tendency of the insured persons to take care of themselves in the private sector. The evidence is that 90% of MHI's expenditures go to this sector [5]. In addition, non-compliance to the NRT is the number one cause of dissatisfaction of insured persons in NAHI's barometer established in 2011 on access to care for beneficiaries of the MHI scheme [7]. For the revision of medical services tariffs, the problem of coordination between the parties that had signed the national convention is real because the notion of convention has become an economy in current use [8]. The aim of this article is to explore the behavior of liberal doctors with regard to their adherence to the National Convention of MHI and thus to propose improvements to make healthcare professionals more adherent to this convention.

## Methods

This article analyzed a cross-sectional survey that was conducted by our team for identifying the knowledge, attitudes and practices in liberal doctors with regard to the national convention signed between the representatives of these doctors and the funds of MHI. The survey was conducted to clarify the barriers and obstacles to the renewal of this national convention which should be renewed 3 years after the start of the MHI plan in 2006 and which has not been renewed until today in 2017. The descriptive field survey was performed for 3 months within the year of 2016. The type of survey was an evaluation of knowledge, attitude and practice known as "KAP survey". Knowledge refers to the act of having information and an adequate understanding of the concept, objectives and activities of MHI without forgetting the benefits and responsibilities associated with it [9]. The attitude refers to the evaluation of emotions with a certain level of positivity or negativity [9]. The practice invokes the adoption of the concept, objectives, responsibilities, role and benefits of the national convention [9]. The objective is to assess the level of knowledge, as a determinant of attitudes that motivate practices [10]. Certain factors can generally influence knowledge, attitude and practice [11]. The factors selected for this type of investigation were age, gender, medical specialty, job tenure, marital status and area of practice. The survey included a population of liberal generalist and specialist doctors who are adhering to the national convention signed under the plan of MHI in Morocco. For the selection of the target population, we applied a random sampling. Following a qualitative approach aimed to explore opinions and behavior, the data collection process was inductive [12]. In addition, the collection tool used is a questionnaire composed, in the majority, of closed questions with single or multiple choice. In Rabat-Sale-Kenitra region, we administered a face-to-face questionnaire with the persons concerned. However, for targets located outside this region, we used a self-administered electronic questionnaire through the "Askabox.fr" website and via the "Gmail" e-mail network. We carried out the collection of emails through the exploitation of the database on the websites "www.santeaumaroc.com" and "www.docteur-contact.com". We presented the questionnaires under the principles of volunteering and anonymity to avoid the bias of social desirability. The survey database is stored and presented in an Excel file. We carried out the processing of the collected data and their analysis by SPSS software 13.0. We expressed all answers of questions in the questionnaire as a number and percentage. We evaluated the relation between the qualitative variables using the chi^2^ test. The p is significant at a value less than 0.05.

## Results

The questionnaire affected 120 doctors; we included in the analysis only 40 questionnaires well filled, ie we have had a response rate of 33%. The majority of doctors surveyed (65%) were between 25 and 50 years old. In our population surveyed, men represented 67.5% and married doctors represented 75%. Three-quarters (75%) of the respondents were specialist doctors and two-thirds (65.8%) have a professional experience of less than 10 years. The response rate observed was higher in the two regions of Rabat-Sale-Kenitra (37.5%) and Casablanca-Settat (30%) among the six-targeted regions. Table 1 summarizes Socio-demographic characteristics of the liberal doctors surveyed. Of the physicians surveyed, 87.5% never participated in training on the tariffs set by the national convention signed between the liberal doctors and the health insurance funds. 97.5% were not satisfied with the tariffs set by the national convention and 100% of the doctors questioned wanted a change in the methods of calculating these tariffs. For the majority (92.5%), the best mode of remuneration was the fee-for-service payment. The results of the study showed that the most important reasons for non-adherence to the national convention were the low level of remuneration, the disorganization of work, the deterioration of working conditions and the scarcity of development prospects. Moreover, the majority (92.5%) considered that health professionals did not respect the national reference tariffs and that the latter does not allow professionals to provide good quality care more specifically with regard to equipment and conditions of work. 95% considered that reference tariffs should be upgraded and 85% considered that their union representativeness was not effective in defending their interest. Regarding the practices of the liberal doctors, the majority followed the recommendations of good practices in their prescriptions and none follows the orientations of the NAHI in its daily practice. 80% of the doctors applied a tariff between 15 and 30 Euros and 65% considered that the exchanges that exist between them and the health insurance funds were weak. 72.5% considered that the complexity of the procedures was a hindrance to the renewal process of the national convention. Table 2 and Table 3 summarize knowledge, attitudes and practice of liberal doctors with regard to the national convention.

**Table 1:** Socio-demographic characteristics of the liberal doctors surveyed

Variables	Modalities	Number	%
**Age**			
	Under 25	4	10
	Between 25 and 50 years	26	65
	More than 50 years	10	25
	Total	40	100
**Gender**			
	Male	27	67.5
	Female	13	32.5
	Total	40	100
**Marital status**			
	Single	8	20
	Married	30	75
	Divorced	2	5
	Total	40	100
**Professional status**			
	General practitioner	8	25
	Specialist	24	75
	Total	32	80
**Professional experience**			
	Less than 5 years	12	31.6
	Between 5 and 10 years	13	34.2
	Between 11 and 20 years	6	15.8
	More than 20 years	7	18.40
	Total	38*	95
**Area of practice**			
	Tangier-Tetouan-Al Hoceima	3	7.5
	Oriental	1	2.5
	Fez-Meknes	5	12.5
	Kenitra-Rabat-Sale	15	37.5
	Marrakech-Safi	4	10
	Greater Casablanca-Settat	12	30
	Total	40	100

* Missing values following voluntary refusal of the participant (explanation unknown)

**Table 2:** Knowledge and attitudes of the liberal doctor contracted to the National Convention

Questions	Yes	%	No	%	I don’t know	%
Participation in a training course on the MHI's agreement	5	12.5	35	87.5	0	0
Satisfaction with the MHI’s NRT	0	0	39	97.5	1	2.5
Change in the calculation of NRT	40	100	0	0	0	0
**Preferred payment method**						
Fee-for-service	37	92.5	1	2.5	2	5
Flat tariff pay	6	15	32	80	2	5
**Non-membership factors**						
Unfair remuneration	32	80	7	17.5	1	2.5
Non-recognition of work	12	30	27	67.5	1	2.5
Deteriorating conditions of exercise	11	27.5	28	70	1	2.5
Lack of prospective for development	13	32.5	26	65	1	2.5
**Acceptability of MHI stakes**						
Compliance with the NRT since 2006	1	2.5	37	92.5	2	5
Access to private care under MHI	14	35	24	60	2	5
Quality of private care under MHI	3	7.5	35	87.5	2	5
Need for upgrading NRT	38	95	0	0	2	5
Effectiveness of union representation	4	10	34	85	2	5

**Table 3:** Practices of the liberal doctor contracted to the National Convention

Questions	Yes	%	No	%	I don’t know	%
Involvement in the sectoral health strategy	12	30	15	37.5	13	32.5
**Determinants of the choice of prescription**						
Experience with patients	32	80	8	20	0	0
Recommendations of good practices	37	92.5	3	7.5	0	0
NAHI guidance	0	0	40	100	0	0
**Usual consultation fee**						
<150DH	5	12.5	35	87.5	0	0
Between 150 and 300 DH	32	80	8	20	0	0
≥ 300 DH	3	7.5	37	92.5	0	0
Frequent exchange of information with MHI	14	35	26	65	0	0
Suggested improvement of the proposed services offered in the framework	3	7.5	29	72,5	8	19.5
Risk of non-reimbursement by the MHI in the third-party payment mode	7	17.5	24	60	9	22.5
Using instruments issued by NAHI	19	47.5	8	20	13	32.5
**Obstacles to the application of the Convention**						
Lack of motivation	24	60	15	37.5	1	2.5
Lack of time	4	10	35	87.5	1	2.5
Lack of educational materials	24	60	15	37.5	1	2.5
**Brakes in the renewal process of the convention**						
Cost of renewal of the agreement	14	35	22	55	4	10
Physician Habits and Behaviors	7	17.5	29	72.5	4	10
Risk of non-compliance	18	45	18	45	4	10
Complexity of procedures	24	60	12	30	4	10

## Discussion

In terms of knowledge, 87.5% of the liberal doctors surveyed were not trained in MHI regulatory tools. In 2015, an annual training plan has been set up for 15,700 generalist and specialist doctors [13]. This action seems to have affected only 12.5% of the total subjects of the survey. In 2016, the NAHI launched an annual training plan that would reach all health professionals [14]. However, the only category that benefited from this training was the pharmacists under the convention of the third-party payment [14]. These figures highlight a major communication problem on the one hand between health professionals and the management bodies of MHI and on the other hand between health professionals and their representativeness. The attitude of the doctors surveyed shows dissatisfaction (97.5%) in particular because of the NRT. This pricing has been the subject of several criticisms and several meetings between the representatives of the liberal doctors and the funds of MHI under the dome of the NAHI in order to be able to improve it and to adapt it to the context of the medical practice. All believe that the change in tariffs is essential for a better adhesion of health professionals to the national convention. The majority (92.5%) of doctors surveyed felt that the fee-for-service method remains the best form of remuneration in the liberal sector. This method of payment encourages and motivates doctors to provide more services because it allows them a greater profitability. Most (80%) consider equitable remuneration to be a major incentive for adherence to the national convention and that compliance with the convention requires a revaluation of medical services (95%). Every three years, the tariff readjustment in the national convention should be done [15]. However, the signing of the endorsement n°2 in 2008 is the only renewed action taken [14]. At the practice level, 37.5% of the liberal doctors surveyed reveal that they don't practice their profession in direct relation with the orientations of the public health policy. In fact, contracting with liberal doctors (Public-Private Partnership) aims, above all, to regulate the provision of care for insured persons and guarantee equitable access to health services [6]. All of the liberal doctors surveyed don't follow NAHI's prescription guidelines; it is mainly in relation with the promotion of the prescription of generic medicines [16]. In this sense, NAHI had launched an initial campaign for the promotion of generic medicines because she believes that the generic could bring huge savings to the health insurance scheme. Furthermore, the majority (87.5%) of the liberal doctors does not respect the national reference tariff for the consultations; this situation can increase the tariff of these consultations and consequently the co-payment of the patients. In the private sector, the remaining share paid by the insured persons of the MHI increased from 33.1% in 2010 to 37.2% in 2015, i.e. an increase of 4.1% [5].

According to the results of our study, 65% of the liberal doctors surveyed didn't share information with MHI management bodies in the last 6 months from the start of the survey. Most of them (72.5%) state that the introduction of tariffs, mainly and the clauses of the national convention, globaly, doesn't benefit from a survey of the liberal doctors' opinions before their approbation and application. To meet this challenge of communication, the NAHI launched the National Integrated Management and Information System (SNIGI) project for basic medical coverage [17]. According to our study, only 47.5% of the liberal doctors surveyed know how to use NAHI's regulatory tools in their daily practices. These tools are references for health professionals and guides for reimbursable medicines and medical devices [13]. The references include, first, a list of standardized MHI documents and secondly, the standardization of certain administrative procedures facilitating communication with MHI management bodies and proper maintenance of repayment records [13]. For the guide to reimbursable medicines, it is an accompaniment to the pharmaceutical policy in order to promote the prescription of generic medicines [13]. Lastly, the majority of respondents (60%) claim that the obstacles to the application of the national convention were, on the one hand, a lack of motivation and on the other hand, a lack of educational materials in correlation with the non-conformity of their practices with the clauses of the national convention. For the renewal of the national convention, the majority (60%) of the liberal doctors surveyed revealed that the complexity of the procedures is a brake in this process. The renewal should involve all stakeholders, who must first establish a climate of mutual trust to understand the constraints of each of the parties. In financial terms, MHI's budgetary position is comfortable for a renewal of the national convention. It is a cumulative surplus of 2.61 billion Euros by the end of 2015 [18]. This financial balance will be maintained at the National Fund of Social Security (NFSS) until 2025, however, for the National Fund for Social Welfare Institutions (NFSWI), actuarial studies predict an initial deficit estimated at 30 million Euros for the year 2016 [13]. In administrative terms, with the conclusion of a new national convention, the NAHI intends to make the remuneration modalities standardized and equitable for liberal doctors in collaboration with the NFSS and the NFSWI [17]. It was also anticipated, since 2015, that the duration of the conventions will be flexible in order to unlock the negotiations on the NRT over time [17]. Other interventions include making the negotiation of national conventions sectoral with doctors, separating the agreement between health professionals and healthcare institutions, the legitimizing the negotiating authorities [17]. The NAHI, regulator of health insurance schemes, therefore plans to resort to certain actions for the renewal of the national convention through a more effective model. However, this model must take into account certain findings in the common behavior of the liberal doctors in Morocco.

Patient access to care in the public sector follows the principles of continuity, equality and adaptation [19]. Physicians in this sector are more interested in raising the general budget of their institutions as a source of funding [20]. They accept MHI policyholders by applying common tariffs and streamlining their behavior while trying to improve the quality of care [21]. On the other hand, in liberal medicine, the principles are the freedom of installation of doctors, the freedom of prescription, the free fixing of fees (direct agreement between doctor and patient) and direct payment of fees [22]. These principles run counter to the logic of public health care and social protection provided by MHI. In the national convention, the liberal doctor, while respecting its principles, must therefore play a role in providing information on household health expenditures, efficiency of spending (appropriate care and no overconsumption) and access to care of patients. The freedom of prescription for the Moroccan liberal doctor finds its limit in the conditions of the patients [22]. The physician is aware that he must never give a patient a medication of lower quality, safety or effectiveness for profit, taking advantage of the asymmetry of information that exists between the patient and the doctor [22]. In this context, the prescription of generic medicines must reassure the patient about the quality of these drugs and their bioequivalence with respect to originator medicines [23]. The national convention must also protect the freedom of installation, tariffs fixing and direct payment for the liberal doctor [24]. As such, it would be pertinent to link the practice of a competing tariff with various regulatory incentives accompanied by free membership of the national convention [25]. As the French experience illustrates, the behavior of the liberal doctors reveals a certain level of fee overruns that remain even with the obtaining of their direct agreement for the NRT [25]. Nevertheless, these exceedances will have to be controlled so that they do not impoverish the patient. There could be an adoption, for example, of the logic of the sectors as in France or a strengthening of the application of sanctions [25]. Medical ethics will also need to be promoted among these liberal doctors to take more account of the financial situation of their clients [26]. Controlling fee overruns obviously requires the collaboration of these health professionals [27]. In Morocco, this involves the SNIGI, which allows electronic reporting of medical activities that are monitored by the dashboards. The limits of our work are mainly represented by the small size of our sample, especially of those who have accepted to complete the questionnaire, which leaves any extrapolation to the entire liberal medical community quite difficult. The problem consists in the liberal doctors who refuse to declare of their non-adherence to the convention even if they do not respect the national reference tariff.

## Conclusion

The study shows that there is some limits to apply and respect the clauses of the national convention between liberal doctors and health insurance funds. The national convention should allow the satisfaction of the liberal doctors and should win their commitment to the principles of the MHI while mastering the financial equilibrium of the funds and the financial accessibility of the insured. The acceleration of the renewal of the national convention can only be achieved through the commitment of doctors to collaborate to finalize its administrative procedures with a view to rationalizing expenditure. The negotiations around the national convention between the liberal doctors and the MHI management and regulatory bodies reveal a confrontation of powers and interests. This type of negotiation would be complex and difficult to manage without an in-depth study of problems such as the influence of actors' representations on power sharing and control of information as a source of power. we suggest to conduct others studies for having a large sample and representative nationwide in order to have a more interesting results.

### What is known about this topic

- Around the world, the health insurance plan signs agreements with healthcare professionals to define the rules for reimbursing the benefits provided to insured persons and especially to define the reimbursement tariffs for these benefits;
- The respect of the agreements signed in the framework of the health insurance by the doctors represents a challenge for the national authorities;
- Several countries have adopted bonus or penalty systems for doctors who respect convention tariffs or who do not respect them.

### What this study adds

- The results of our study showed that the attitudes of health professionals towards the national agreement signed under the health insurance scheme tend towards the non-respect of the national reference tariffs because they consider that these tariffs are low and do not reflect the volume and quality of the services they provide to the insured;
- This situation has a negative impact on the purchasing power of policyholders because they will have to pay a high user fee, which represents the difference between the national reference tariff refunded by the health insurance funds and the tariff applied by the liberal doctors;
- It is important for health insurance plan partners to sit down and try to review the terms of the agreement and increase reference tariffs in order to minimize policyholder co-payments and enable the viability of healthcare structures while maintaining the balance of health insurance funds.
